# Clinical characteristics of IgG4-related retroperitoneal fibrosis versus idiopathic retroperitoneal fibrosis

**DOI:** 10.1371/journal.pone.0245601

**Published:** 2021-02-18

**Authors:** Kunkun Wang, Zhenfan Wang, Qiaozhu Zeng, Lijuan Zhu, Jingyuan Gao, Ziqiao Wang, Shanshan Zhang, Fei Yang, Danhua Shen, Yi Wang, Yanying Liu

**Affiliations:** 1 Department of Rheumatology and Immunology, Peking University People’s Hospital, Beijing, China; 2 Department of Rheumatology and Immunology, Tengzhou Central People’s Hospital, Tengzhou, China; 3 Department of Rheumatology and Immunology, Zhengzhou Central Hospital Affiliated to Zhengzhou University, Zhengzhou, China; 4 Department of Geriatrics, Affiliated Hospital of North China University of Technology, Tangshan, China; 5 Department of Ultrasound, Peking University People’s Hospital, Beijing, China; 6 Department of Pathology, Peking University People’s Hospital, Beijing, China; 7 Department of Radiology, Peking University People’s Hospital, Beijing, China; Leiden University Medical Center, NETHERLANDS

## Abstract

Retroperitoneal fibrosis (RPF) is an uncommon condition characterized by inflammation and fibrosis in the retroperitoneal space. More than two-thirds of RPF are idiopathic, with the remaining stemed from a variety of secondary causes. It was suggested that IgG4-related RPF is a secondary form of RPF. We undertook this study to compare detailed demographic, clinical and laboratory characteristics of IgG4-related RPF and IRPF in a large Chinese cohort. We retrospectively reviewed the medical records of 132 RPF patients diagnosed at Peking University People’s Hospital between March 2010 and March 2018. Among the 132 patients, the mean age at disease onset was 54.8 years. IgG4-related RPF group showed greater male predominance compared to IRPF group. IgG4-related RPF patients showed a longer interval between symptom onset and diagnosis, and allergic diseases were more common in this group. Sixty-four patients (48.4%) had lower back pain, which was more common in IRPF group than that in IgG4-related RPF patients. In terms of organ involvement, although 42 of 47 patients (89.3%) with IgG4-related RPF had other organ involvement, there were no patients in the IRPF group with other organ involvement. In addition, the serum IgG4 level, elevated eosinophils counts and IgE level were significantly higher in IgG4-related RPF patients. We described the demographic, clinical and laboratory differences between IgG4-related RPF and IRPF patients, indicating their potential differences in pathogenesis, which was of great importance to diagnose and manage the two phenotypes.

## Introduction

Retroperitoneal fibrosis (RPF) is a rare disorder characterized by the presence of chronic inflammation and fibrosis in the retroperitoneal space [1]. It usually involves the adventitia of the abdominal aorta, the iliac arteries and the adjacent structures, which results in flank pain, lower extremity edema, hydronephrosis, ureteral obstruction and other clinical symptoms [2]. More than two-thirds of RPF are idiopathic, with the remaining stemed from secondary causes, including infections, trauma, tumor, autoimmune diseases, drugs, radiation therapy, and surgery [3, 4].

Last decade, the concept of IgG4-related disease (IgG4-RD) emerged. IgG4-RD consists of fibro-inflammatory diseases that affect a variety of structures (e.g, pancreas, biliary tract, lymph nodes, retroperitoneum). It is featured with lympho-plasmacytic inflammation, infiltration by IgG4^+^ plasma cells and irregular and distinct fibrosis [5, 6]. It was recently suggested that IgG4-related RPF is a secondary form of RPF, and thus it is included in the IgG4-related spectrum of sclerosing disease [7–10].

Researchers have proposed that the pathogenesis of IgG4-related RPF may be different with that of idiopathic retroperitoneal fibrosis (IRPF) [11–13]. However, Choi [14] found that the clinical and laboratory characteristics of IgG4-related RPF are similar to those of IRPF, except for male predominance, older age, and higher incidence of postrenal AKI in IgG4-related RPF. Therefore, we conducted this study to compare the demographic, clinical and laboratory characteristics of IgG4-related RPF and IRPF patients in a large Chinese cohort. Defining the characteristics of these two groups might help facilitate advanced recognition of this condition, identify its risk factors and develop specific treatment strategies for each cluster.

## Materials and methods

### Patients

We retrospectively reviewed the medical records of RPF patients at Peking University People’s Hospital, from March 2010 to March 2018. A total of 132 cases were diagnosed with RPF, and RPF from other secondary causes were excluded, such as malignancy, infection, drugs, surgical injury, and radiation therapy. Patients who did not receive serum IgG4 detection were also excluded. The diagnosis of RPF was based on the reports of Computed tomography (CT) or magnetic resonance imaging (MRI) from radiologists. Well-delimited but irregular soft-tissue mass surrounding the aorta, which extends from the renal arteries to the iliac vessels was deemed as typical manifestation of image of RPF [11]. Retroperitoneal biopsies were not routine, which were usually performed when atypical localization was involved or the clinical feature was highly suggestive of malignancy. Allergic disease was diagnosed according to the criteria from the European Academy of Allergy and Clinical Immunology (EAACI) by experienced specialists. All patients in the study were followed up until death or 31 March 2019.

Patients were divided into two groups, IgG4-related RPF and IRPF, based on the comprehensive diagnostic criteria published by Umehara et al [15] in 2011. Patients with RPF of unknown origin are labelled as having IRPF [1]. This study was approved by the Institutional Review Board of Peking University People’s Hospital (Beijing, China). All authors did not have any access to information that could identify individual participants during or after data collection.

### Clinical and laboratory data

A standardized case report form was used for data collection. Age at disease onset, age at diagnosis, gender, follow up time, clinical symptoms, smoking history, biopsy status, complication of allergic disease were included in the forms. The location of the retroperitoneal fibrotic mass was classified as periaorta, renal pelvis and ureter by image. For patients who had undergone biopsy, the pathology reports were reviewed.

Laboratory index comprised hemoglobin, eosinophil count, C-reactive protein (CRP), erythrocyte sedimentation rate (ESR), serum creatinine before the initial treatment, complement 3 (C3), C4, immunoglobulin E (IgE), immunoglobulin G4 (IgG4) and autoantibodies.

According to literature, the treatment response of IgG4-related RPF was defined as improvement in symptoms attributed to RPF or the dimensions of the imaging finding [16–18]. And the relapse of IgG4-related RPF was defined as deterioration of clinical symptom, organ dysfunction, or radiological examination [19].

### Statistical analysis

All statistical analyses were performed by SPSS 24.0. Data were analyzed using descriptive methods, including mean ± SD, median, interquartile range (IQR) and proportions. The Student’s *t*-test was used to analyze differences for continuous, normally distributed data; and continuous, non-normally distributed data were analyzed with the Mann–Whitney test. Chi-square or Fisher’s exact tests, as appropriate, was used to process categorical variables. It was considered statistically significant when a *P*-value was less than 0.05.

## Results

### Patients’ demographic characteristics

A total of 132 patients diagnosed as RPF were enrolled in this study. Among the 132 cases in our cohort, 47 patients (35.6%) were classified as having IgG4-related RPF (19 definite, 5probable, 23possible), and 85 patients were classified as having IRPF. The demographic features of all cases are shown in Table 1. The mean age at disease onset was 54.8 years, and 70% patients were male. Sixty-four patients (48.4%) underwent biopsies. Among them, 24 cases were identified as IgG4-related RPF, and 40 cases were identified as IRPF. Established allergic diseases were found in fourteen cases (10.6%). Cardiovascular disease risk factors, i.e. hypertension, diabetes mellitus, and smoking, were found in 40 (30.3%), 21 (15.9%), and 60(45.4%) patients, respectively.

**Table 1:** Demographic and clinical characteristics of 132 RPF patients.

Characteristics	Value
Number of cases, n	132
Gender (Male: Female)	2.38:1
Mean age at disease onset (years±SD)	54.8±12.4
Mean age at diagnosis (years±SD)	56.8±12.6
Time from onset to diagnosis, median (IQR)	15(6–26)
Follow up time (months), median (IQR)	20(12–48)
Biopsy, n (%)	64(48.4)
Complication of allergic disease, n (%)	14(10.6)
Hypertension, n (%)	40 (30.3%)
Diabetes mellitus, n (%)	21 (15.9%)
Smoking, n (%)	60(45.4)

We compared the demographic features of patients with IgG4-related RPF against cases of IRPF in Table 2. The gender ratio (Male:Female) of IgG4-related RPF group and IRPF group were 4.8:1 and 1.7:1, respectively (*P* = 0.012). The time from disease onset to diagnosis was 26 months in IgG4-related group, which was significantly longer than that in IRPF group (12 months) (*P* = 0.001). The mean follow-up time of IgG4-related RPF group and IRPF patients was 20 months and 24months, respectively. In terms of allergic disorders, IgG4-RD RPF patients (21.2%) had a higher frequency of allergic diseases (allergic rhinitis, urticaria, asthma) than that in IRPF patients (4.7%) (*P* = 0.006).

**Table 2:** Differences in demographic characteristics between IgG4-related RPF and IRPF patients.

Characteristics	IgG4-related RPF	IRPF	*P*-value
Number of cases, n	47	85	
Gender (Male: Female)	4.8:1	1.7:1	0.012
Mean age at disease onset (years±SD)	55.7±12.0	54.3±12.7	0.532
Mean age at diagnosis (years±SD)	59.4±12.3	55.4±12.7	0.082
Time from onset to diagnosis, median (IQR)	26(14–38)	12(6–16)	0.001
Follow-up time (months), median (IQR)	20(12–36)	24(12–48)	0.251
Complication of allergic disease, n (%)	10(21.2)	4(4.7)	0.006
Biopsy, n (%)	24(51%)	40(47%)	0.659

### Clinical features

**Fig 1:**
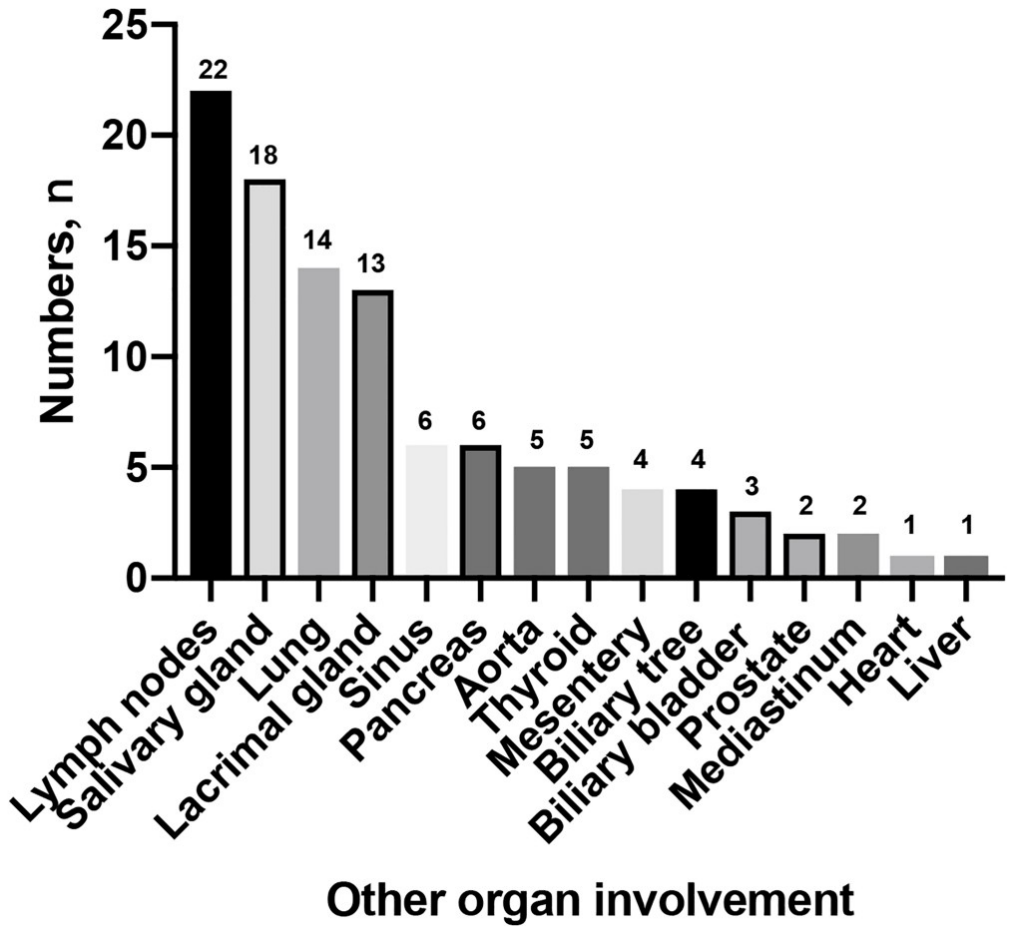
The type of the other organ involvement in IgG4 related RPF patients.

Clinical symptoms varied from patient to patient. Pain was the most common presenting symptom. Sixty-four patients (48.4%) had lower back pain, which was more common in IRPF group (*P* = 0.035). Thirty-five patients (26.5%) had flank pain and twenty-seven (20.4%) had abdominal pain, respectively. Hydronephrosis occurred in 85 of 132 patients (64.3%), with 35 bilateral and 50 unilateral involvement, however, there was no statistical difference between this two groups. Other constitutional symptoms were fever, weight loss, lower limb edema, venous thrombus, and anorexia (Table 3). Organ involvement was evaluated by systematic standards including symptoms, signs, radiographic or other image examinations. Although 42 of 47 patients (89.3%) with IgG4-related RPF had other organ involvement, with the pancreas being the most commonly affected organ, there were no patients in the IRPF group with other organ involvement (*P*<0.001). The other organ involvement was shown in Fig 1. However, there was no significant difference in incidence of mass location (*P* = 0.482).

**Table 3:** Differences in clinical characteristics between IgG4-related RPF and IRPF patients.

Characteristics	Total	IgG4-related RPF	IRPF	*P*-value
Clinical symptom				
Abdominal pain, n (%)	27(20.4)	12(25.5)	15(17.6)	0.282
Flank pain, n (%)	35(26.5)	15(31.9)	20(23.5)	0.296
Lower back pain, n (%)	64(48.4)	17(36.1)	47(55.2)	0.035
Lower limb edema, n (%)	12(9.0)	5(10.6)	7(8.2)	0.754
Anorexia, n (%)	6(4.5)	2(4.2)	4(4.7)	0.905
Weight loss, n (%)	8(6.0)	5(10.6)	3(3.5)	0.132
Fever, n (%)	7(5.3)	2(4.2)	5(5.8)	1.000
Oliguria or anuria, n (%)	6(4.5)	3(6.3)	3(3.5)	0.666
Venous thrombus, n (%)	2(1.5)	1(2.1)	1(1.1)	1.000
Bilateral hydronephrosis, n (%)	35(26.5)	16(34.0)	19(22.3)	0.145
Unilateral hydronephrosis, n (%)	50(37.8)	19(40.4)	31(36.4)	0.654
Mass location				
Aorta, n (%)	56(42.4)	22(46.8)	34(40)	0.482
Renal pelvis, n (%)	11(8.3)	5(10.6)	6(7.0)	
Ureter, n (%)	65(49.2)	20(42.5)	45(52.9)	
Other organ involvements n (%)	42(31.8)	42(89.3)	0(0)	<0.001

### Laboratory findings

We further compared the laboratory tests between these two groups (Table 4). The levels of inflammatory markers such as ESR and CRP were not statistically different between groups, while the number of patients with elevated CRP was significantly higher in the IRPF group (61.1% vs 42.5%, *P* = 0.04). Hypocomplementemia was a common finding in both IgG4-related RPF (29.8%) and IRPF group (22.4%) (*P* = 0.345), and the number of patients with low serum C4 was significantly higher in IgG4-related RPF group (*P* = 0.024). The serum IgG4 level and IgE level were significantly higher in IgG4-related RPF group. IgG4-related RPF patients were more commonly associated with elevated eosinophils counts (23.4% vs 7.05%, *P* = 0.007).

**Table 4:** Laboratory findings in IgG4-related RPF and IRPF patients.

Parameter	Total	IgG4-related RPF	IRPF	*P*-value
ESR (mm/h), median (IQR)	31(13–61)	39(18–72)	30(12–57)	0.186
Elevated ESR, n (%)	91(68.9)	35(74.4)	56(65.8)	0.307
CRP (mg/L), median (IQR)	9(3–19)	5.93(2.4–16.2)	10(3.15–20)	0.075
Elevated CRP, n (%)	72(54.5)	20(42.5)	52(61.1)	0.040
Eosinophilia, n (%)	17(12.8)	11(23.4)	6(7.05)	0.007
Anemia, n (%)	69(52.2)	26(55.3)	43(50.5)	0.602
Serum creatinine at onset (umol/L)	95(73–130)	90(65–120)	100(74.5–160)	0.145
Hypocomplementemia, n (%)	33(25.0)	14(29.8)	19(22.4)	0.345
Reduced serum C4, n (%)	16(12.1)	11(23.4)	5(5.8)	0.003
Reduced serum C3, n (%)	25(18.9)	8(17.0)	17(20.0)	0.676
Serum IgG4 level (mg/dL), median (IQR)	67(26–203)	300(171–877)	35.8(20–70)	<0.001
Serum IgE (IU/ml), median (IQR)	64(40–157)	98.8(69–277)	50(24.5–99.5)	<0.001

### Treatment

Treatment strategies are reported in Table 5. However, there was no significant difference in both groups. As shown, fifteen patients (11.4%) received no treatment, whereas other patients received medical therapy (31.8%), surgical therapy (9.1%), or both (47.7%). Glucocorticoids are the first-line medical therapy, with initial doses of 0.75–1mg/kg per day of prednisone in our study. About 79.5% of all our patients were initially treated with prednisone. Immunosuppressants used in our cohort comprised cyclophosphamide (CYC) alone in 46 patients (34.8%), Methotrexate (MTX) alone in 3(2.2%) patients, and mycophenolate mofetil (MMF) in twenty (15.1%) patients. Eleven patients (8.3%) experienced immunosuppressant replacement during the period, because of either side effects or poor efficacy. Two patients received rituximab combined with glucocorticoids, due to disease relapse. Tamoxifen was used alone in twelve (9.0%) patients.

**Table 5:** Type of medical and surgical therapy in IgG4-related RPF and IRPF patients.

Type	Total	IgG4-related RPF	IRPF	P-value
No treatment, n (%)	15(11.4)	5(10.6)	10(11.7)	0.845
Medical therapy alone, n (%)	42(31.8)	18(38.2)	24(28.2)	0.235
Surgical therapy alone, n (%)	12(9.1)	2(4.2)	10(11.7)	0.211
Combination (medical and surgical therapy), n (%)	63(47.7)	22(46.8)	41(48.2)	0.875
GCs only, n (%)	11(8.3)	5(10.6)	6(7.0)	0.520
GCs+Tamoxifen, n (%)	12(9.0)	1(2.1)	11(12.9)	0.055
GCs+cyclophosphamide, n (%)	46(34.8)	18(38.2)	28(32.9)	0.536
GCs+Mycophenolate Mofetil, n (%)	20(15.1)	10(21.2)	10(11.7)	0.144
GCs+Methotrexate, n (%)	3(2.2)	1(2.1)	2(2.3)	0.934
GCs+Rituximab, n (%)	2(1.5)	1(2.1)	1(1.1)	0.668
GCs+two or more immunosuppressants, n (%)	11(8.3)	4(8.4)	7(8.2)	0.956
Percutaneous nephrostomy, n (%)	14(10.6)	2(4.2)	12(14.1)	0.137
Ureterolysis, n (%)	18(13.6)	4(8.4)	14(16.4)	0.234
Ureteral stents, n (%)	55(41.6)	16(34.0)	39(45.8)	0.257
Resection of retroperitoneal mass, n (%)	6(4.5)	2(4.2)	4(4.7)	0.905

GCs = glucocorticoids.

With regard to surgical interventions, 55 patients (41.6%) had ureteral stents to help alleviate the symptoms. Ureterolysis was carried out in 18 of 132 patients. Fourteen patients (10.6%) underwent percutaneous nephrostomy.

We further examine the role of serum IgG4 level in treatment among IgG4-related RPF. Among 47 patients with IgG4-related RPF, 34 cases (72.3%) achieved treatment response and 10 patients (21.3%) showed relapse during the follow-up period. The median serum IgG4 level of response and no response group were 252 (171–877) mg/dl and 403 (210–669) mg/dl, respectively (*P* = 0.868). And the median serum IgG4 level of relapse and no relapse group were 581.5 (243–959) mg/dl and 244 (162–669) mg/dl, respectively (*P* = 0.082).

## Discussion

We compared herein the demographic, clinical and laboratory features between 47 IgG4-related RPF patients and 85 IRPF patients, differences in the above aspects were found. To our knowledge, this is the first research on such two phenotypes of RPF in China, and the sample size is large.

In our current study, male predominance has been described in both groups. However, IgG4-related RPF group showed greater male predominance compared to IRPF group. This is comparable to several previous studies [14, 20–22]. It was found that IRPF patients got a diagnosis at an earlier age than IgG4-related RPF patients, although the comparison between the two groups fell short of statistical significance. In 2019, Maritati et al. [23] conducted a retrospective observational study including 113 chronic periaortitis (CP) patients, which comprised IRF, IgG4-RPF, and inflammatory abdominal aortic aneurysms patients. They also found an earlier age at diagnosis in CP patients with normal serum IgG4 level. In our study, more patients in IRPF group complain lower back pain initially than that in IgG4-related RPF, which is easily noticed even in the absence of other symptoms. Maybe it could explain time from onset to diagnosis of IRPF patients was shorter than that in IgG4-related RPF patients. Moreover, IgG4-related RPF is a newly known disease, and it takes time for doctors to identify it.

Most reports of IgG4-related RPF describe extraperitoneal involvement of IgG4-related disease with RPF [9, 22, 24]. Sialadenitis, autoimmune pancreatitis (AIP), periaortitis of the thoracic aorta, and reticular lung lesions can exist simultaneously, while other lesions are not identified in IRPF patients. Similar to previous reports [12, 21], other organ involvements were much more frequent in IgG4-related RPF (89.3% vs. 0%).

IgG4-related RPF patients tended to manifest with eosinophilia, higher serum IgG4 level, higher IgE level as well as allergic condition. Such findings indicated different pathogenesis between these two phenotypes. Recent evidences suggested that high serum IgG4 levels was linked to IgG4-related RPF [1, 22], and T-helper 2 (Th2)-polarization was observed in IgG4-related RPF [25]. Therefore, we assumed that the higher serum IgG4 levels in IgG4-related RPF patients was caused by the Th2 cytokines such as interleukin-4 and interleukin-13, which enhance the production of both IgG4 and IgE [26]. Additionally, these cytokines may enrich the IgG4+ plasma cell [27]. Eosinophilia and elevated serum IgE levels, both observed in approximately 40% of patients with IgG4-related disease, are also mediated by Th2 cytokines [3, 28]. We consider that our finding could strength the hypothesis that IgG4-related RPF is induced preferentially in the setting of a Th2-cell dominant immune reaction.

CRP levels are increased in the majority of patients in our cohort (54.5%), which is consistent with previous studies [27, 29]. The proportion of elevated CRP patients in IRPF group was higher than that in IgG4-related RPF group. CRP is often used to monitor the clinical course of the disease, though they do not always reliably mirror disease activity [30]. Additionally, more research is needed to confirm whether IRPF patients is more active in inflammatory response.

Steroid therapy is the main treatment option for IgG4-related and IRPF patients. Although there are currently no standard treatment regimens, moderate to high dose of steroids with slow tapering are recommended [31, 32]. In our study, tamoxifen and immunosuppressants (cyclophosphamide, mycophenolate mofetil, and methotrexate) are used as glucocorticoid-sparing agents or remission-maintenance drugs. For patients with recurrent or refractory disease, B-cell depletion with rituximab appears to be a useful approach [18]. With regard to patients with ureteral compression, the first goal of treatment is the relief of ureteral obstruction [2]. In this study, no significant relationship between serum IgG4 level and treatment response or relapse were found in IgG4-related RPF patients, although no response group and relapse group tended to have a relatively higher serum IgG4 level, which was consistent with our previous research [33]. Actually, it is controversial with respect to the role of serum IgG4 level in assessing disease activity and treatment outcome. Monitoring of IgG4 concentration is considered to be useful in assessment of disease activity only in part of IgG4-RD patients. Specifically, serum IgG4 concentrations do not normalize in nearly 63% of cases after glucocorticoid treatment and do not rise again at disease relapse in nearly 10% of cases [34, 35]. Furthermore, the IgG4-RD Responder Index (RI), a tool to help investigators assess the efficacy of treatment and disease activity, had excluded serum IgG4 concentration as a scoring factor due to the lack of sensitivity and specificity for evaluating disease activity in its latest version [36].

To our knowledge, this is the first study to compare the differences between the two groups in such large sample size. However, this study also has some limitations. This observational study was limited by its retrospective nature with inherent biases, some potential confounding factors could not be well ruled out. And the retroperitoneal biopsies were not conducted for every patient, partly resulting from the fact that retroperitoneal biopsy is a high-risk and invasive procedure.

In conclusion, we have analyzed demographic, clinical and laboratory differences between IgG4-RD RPF and IRPF patients. IgG4-RD RPF patients were associated with longer time from onset to diagnosis and showed greater male predominance compared to IRPF patients. Allergic diseases and multi-organ involvement were more commonly observed in IgG4-RD RPF group. IgG4-RD RPF phenotype showed higher level of IgG4 and IgE. These findings indicated potential differences in pathogenesis and important implications for the diagnosis and management of these two phenotypes.

## Supporting information

S1 File Data set of this study. (XLS)

S1 Checklist STROBE statement—Checklist of items that should be included in reports of observational studies. (DOCX)
