# Supplementary material for: Clinical characteristics of IgG4-related retroperitoneal fibrosis versus idiopathic retroperitoneal fibrosis
Source: PLoS One. 2021 Feb 18;16(2):e0245601. doi: 10.1371/journal.pone.0245601 (PMC7891782; doi:10.1371/journal.pone.0245601)
Supplement: S1 Checklist — (DOCX) [file pone.0245601.s002.docx]

STROBE Statement—checklist of items that should be included in reports of observational studies

|  | Item No. | Recommendation | Page  No. | Relevant text from manuscript |
| --- | --- | --- | --- | --- |
| **Title and abstract** | 1 | (*a*) Indicate the study’s design with a commonly used term in the title or the abstract | 1-2 | Clinical characteristics of IgG4-related retroperitoneal fibrosis versus idiopathic retroperitoneal fibrosis  / We retrospectively reviewed the medical records of 132 RPF patients diagnosed at Peking University People’s Hospital between March 2010 and March 2018. |
|  |  | (*b*) Provide in the abstract an informative and balanced summary of what was done and what was found | 2-3 | Among the 132 patients, the mean age at disease onset…which was of great importance to diagnose and managethe two phenotypes. |
| Introduction | | | |  |
| Background/rationale | 2 | Explain the scientific background and rationale for the investigation being reported | 4 | Researchers have proposed that the pathogenesis of IgG4-related RPF may be…and higher incidence of postrenal AKI in IgG4-related RPF |
| Objectives | 3 | State specific objectives, including any prespecified hypotheses | 4-5 | Therefore, we conducted this study to…specific treatment strategies for each cluster. |
| Methods | | | |  |
| Study design | 4 | Present key elements of study design early in the paper | 5 | We retrospectively reviewed the medical records of RPF patients at Peking University People’s Hospital, from March 2010 to March 2018. |
| Setting | 5 | Describe the setting, locations, and relevant dates, including periods of recruitment, exposure, follow-up, and data collection | 5 | The clinical data used in our study was accessed by March 2020 |
| Participants | 6 | (*a*) *Cohort study*—Give the eligibility criteria, and the sources and methods of selection of participants. Describe methods of follow-up  *Case-control study*—Give the eligibility criteria, and the sources and methods of case ascertainment and control selection. Give the rationale for the choice of cases and controls  *Cross-sectional study*—Give the eligibility criteria, and the sources and methods of selection of participants | 5 | A total of 132 cases were diagnosed with RPF, and RPF from other secondary causes were excluded…Patients who did not receive serum IgG4 detection were also excluded. |
|  |  | (*b*) *Cohort study*—For matched studies, give matching criteria and number of exposed and unexposed  *Case-control study*—For matched studies, give matching criteria and the number of controls per case | NA |  |
| Variables | 7 | Clearly define all outcomes, exposures, predictors, potential confounders, and effect modifiers. Give diagnostic criteria, if applicable | 5-6 | The diagnosis of RPF was based on the reports of Computed tomography (CT) or…are labelled as having IRPF |
| Data sources/ measurement | 8* | For each variable of interest, give sources of data and details of methods of assessment (measurement). Describe comparability of assessment methods if there is more than one group | 6 | A standardized case report form was used for data collection…and autoantibodies. |
| Bias | 9 | Describe any efforts to address potential sources of bias | 20 | However, it was a retrospective research…invasive procedure. |
| Study size | 10 | Explain how the study size was arrived at | 7 | A total of 132 patients diagnosed as RPF were enrolled in this study. |

Continued on next page

| Quantitative variables | 11 | Explain how quantitative variables were handled in the analyses. If applicable, describe which groupings were chosen and why | 7 | The Student’s t-test was used to analyze differences for continuous, normally distributed data; and continuous non-normally distributed data were analyzed with the Mann–Whitney test. |
| --- | --- | --- | --- | --- |
| Statistical methods | 12 | (*a*) Describe all statistical methods, including those used to control for confounding | 7 | All statistical analyses were performed…statistically significant when a P-value was less than 0.05. |
|  |  | (*b*) Describe any methods used to examine subgroups and interactions | NA |  |
|  |  | (*c*) Explain how missing data were addressed | NA |  |
|  |  | (*d*) *Cohort study*—If applicable, explain how loss to follow-up was addressed  *Case-control study*—If applicable, explain how matching of cases and controls was addressed  *Cross-sectional study*—If applicable, describe analytical methods taking account of sampling strategy | NA |  |
|  |  | (*e*) Describe any sensitivity analyses | NA |  |
| Results | | | | |
| Participants | 13* | (a) Report numbers of individuals at each stage of study—eg numbers potentially eligible, examined for eligibility, confirmed eligible, included in the study, completing follow-up, and analysed | 7 | A total of 132 patients diagnosed as RPF were enrolled in this study. All patients in the study were followed up until death or 31 March 2019. |
|  |  | (b) Give reasons for non-participation at each stage | NA |  |
|  |  | (c) Consider use of a flow diagram | NA |  |
| Descriptive data | 14* | (a) Give characteristics of study participants (eg demographic, clinical, social) and information on exposures and potential confounders | 7 | Table 1 |
|  |  | (b) Indicate number of participants with missing data for each variable of interest | NA |  |
|  |  | (c) *Cohort study*—Summarise follow-up time (eg, average and total amount) | NA |  |
| Outcome data | 15* | *Cohort study*—Report numbers of outcome events or summary measures over time | *NA* |  |
|  |  | *Case-control study—*Report numbers in each exposure category, or summary measures of exposure | *NA* |  |
|  |  | *Cross-sectional study—*Report numbers of outcome events or summary measures | 10-17 |  |
| Main results | 16 | (*a*) Give unadjusted estimates and, if applicable, confounder-adjusted estimates and their precision (eg, 95% confidence interval). Make clear which confounders were adjusted for and why they were included | 10-17 | Table 2-5 |
|  |  | (*b*) Report category boundaries when continuous variables were categorized | NA |  |
|  |  | (*c*) If relevant, consider translating estimates of relative risk into absolute risk for a meaningful time period | NA |  |

Continued on next page

| Other analyses | 17 | Report other analyses done—eg analyses of subgroups and interactions, and sensitivity analyses | NA |  |
| --- | --- | --- | --- | --- |
| Discussion | | | | |
| Key results | 18 | Summarise key results with reference to study objectives | 18 | We compared herein the demographic…differences in the above aspects were found. |
| Limitations | 19 | Discuss limitations of the study, taking into account sources of potential bias or imprecision. Discuss both direction and magnitude of any potential bias | 20 | However, it was a retrospective research and retroperitoneal biopsies…a high-risk and invasive procedure. |
| Interpretation | 20 | Give a cautious overall interpretation of results considering objectives, limitations, multiplicity of analyses, results from similar studies, and other relevant evidence | 20 | We have analyzed demographic…for the diagnosis and management of these two phenotypes. |
| Generalisability | 21 | Discuss the generalisability (external validity) of the study results | 20 | To our knowledge, this is the first study to compare the differences between the two groups in such large sample size. |
| Other information | |  | | |
| Funding | 22 | Give the source of funding and the role of the funders for the present study and, if applicable, for the original study on which the present article is based | NA |  |

*Give information separately for cases and controls in case-control studies and, if applicable, for exposed and unexposed groups in cohort and cross-sectional studies.

**Note:** An Explanation and Elaboration article discusses each checklist item and gives methodological background and published examples of transparent reporting. The STROBE checklist is best used in conjunction with this article (freely available on the Web sites of PLoS Medicine at http://www.plosmedicine.org/, Annals of Internal Medicine at http://www.annals.org/, and Epidemiology at http://www.epidem.com/). Information on the STROBE Initiative is available at www.strobe-statement.org.
